# Is there a Place for Prebiotics in the Management of Neonatal Inguinal Hernia? A Preliminary Study

**DOI:** 10.21699/jns.v6i1.488

**Published:** 2017-01-01

**Authors:** Mahdi Ben Dhaou, Mohamed Zouari, Saloua Ammar, Amira Bouraoui, Imene Gassara, Ines Feki, , Hayet Zitouni, Mohamed Jallouli, Jawaher Masmoudi, Abdellatif Gargouri, Riadh Mhiri

**Affiliations:** 1 Department of Pediatric Surgery, Hedi Chaker Hospital, Sfax, Tunisia; 2 Department of Neonatology, Hedi Chaker Hospital, Sfax, Tunisia; 3 Department of Psychiatry A, Hedi Chaker Hospital, Sfax, Tunisia

**Keywords:** Inguinal hernia, Infant, Surgery, Prebiotic, Colic

## Abstract

The objective of this study was to assess the place of prebiotics in the management of neonatal inguinal hernia. Boys with a diagnosis of unilateral non-complicated inguinal hernia, aged less than 40 days, were prospectively followed from January 2012 to December 2014. Clinical and psychiatric data and outcomes were collected before and after prebiotics (Primalac AC) administration. Ninety-eight patients were included. There were 75 inguinal hernias and 23 inguino-scrotal hernias. Before prebiotics administration 72.2% of infants had abdominal distention and 98% had colic. After prebiotics, abdominal distention and colic regressed in 85.2% and 73.2% of patients, respectively. Hernias disappeared clinically in 66.3% of cases. The factors associated with the disappearance of hernias were the type of the hernia (p<0.001), colic (p<0.001), and abdominal distention (p<0.001). Prebiotics would be a new adjunct in the management of neonatal inguinal hernia. They decrease colic and abdominal distention, which seems helpful to prevent strangulation and probably get spontaneous resolution of small hernias.

Inguinal hernia is a common congenital anomaly. The incidence in children varies between 0.8% and 4.4%. The most serious complication is strangulation [1]. Surgical repair is often recommended shortly after the diagnosis. However, opting for early surgical treatment during neonatal period accounts an anesthetic and surgical challenge. The herniated sack is friable. A recurrence rate of 8.6% has been reported [2]. Perioperative small changes in tidal volume, during mechanical ventilation of neonates, can induce lung injury and excess of oxygen may be harmful [3]. High anesthesia mortality and postoperative apnea rate are reported in many studies from developing countries [4-5]. The objective of this study was to assess the place of prebiotics in the management of neonatal inguinal hernia.

Boys with a diagnosis of unilateral non-complicated inguinal hernia, aged less than 40 days, were prospectively followed from January 2012 to December 2014. Clinical data and outcomes were collected before and after prebiotics (Primalac AC) administration. The daily dose of the prebiotics, route and frequency are illustrated in Table 1. Preoperative diagnosis of hernia was based on clinical history and examination. The resolution of hernia was based on clinical and ultrasonographical findings, and the parents confirming no visible swelling for more than 1 month.

Ninety-eight patients were included. There were 75 inguinal hernias and 23 inguino-scrotal hernias. Before prebiotics administration 72.2% of infants had abdominal distention and 98% had colic. Eighty-five patients were on breastfeeding. After prebiotics, abdominal distention and colic regressed respectively in 85.2% and 73.2% of patients. Hernias regressed in 66.3% of cases and the average time of presumed resolution was 12 weeks (2 to 22 weeks). The factors associated with the regression of hernias were the type of the hernia (p<0.001), colic (p<0.001) and abdominal distention (p<0.001). Breastfeeding was not significantly associated with regression of hernias (p=0.541) (Table 2).

**Figure:**
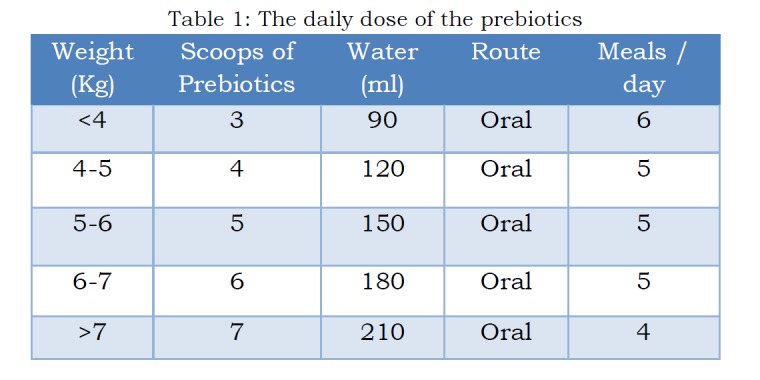
Table 1: The daily dose of the prebiotics.

**Figure:**
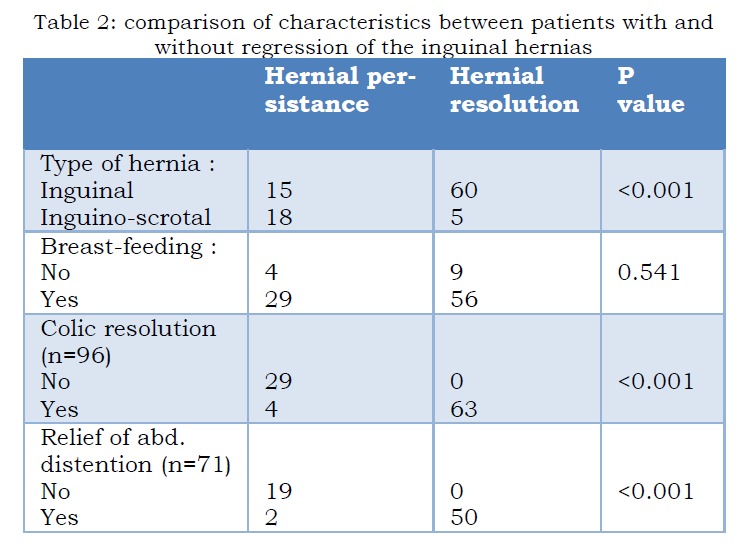
Table 2: comparison of characteristics between patients with and without regression of the inguinal hernias.

In conclusion, prebiotics would be a new adjunct in the management of neonatal inguinal hernia. They decrease colic and abdominal distention, which seems helpful to prevent pushing of gut into the hernial sac and thus may help in spontaneous closure of the hernial sac as happens in hydrocele. Further investigations are needed to validate these findings.

## Footnotes

**Source of Support:** None

**Conflict of Interest:** None
